# Integrin subunit alpha V is a potent prognostic biomarker associated with immune infiltration in lower-grade glioma

**DOI:** 10.3389/fneur.2022.964590

**Published:** 2022-10-25

**Authors:** Zilong Tan, Zhe Zhang, Kai Yu, Huan Yang, Huaizhen Liang, Tianzhu Lu, Yulong Ji, Junjun Chen, Wei He, Zhen Chen, Yuran Mei, Xiao-Li Shen

**Affiliations:** ^1^Department of Neurosurgery, The Second Affiliated Hospital of Nanchang University, Nanchang, China; ^2^Jiangxi Key Laboratory of Translational Cancer Research, Jiangxi Cancer Hospital of Nanchang University, Nanchang, China; ^3^The Graduate Department, Jiangxi Medical College of Nanchang University Nanchang, Nanchang, China; ^4^Department of Neurosurgery, People's Hospital of Wuhan University, Wuhan, China; ^5^Department of Neurosurgery, Changde Hospital of Traditional Chinese Medicine, Changde, China; ^6^Department of Orthopaedics, Union Hospital, Tongji Medical College, Huazhong University of Science and Technology, Wuhan, China; ^7^Department of Radiation Oncology, Jiangxi Cancer Hospital of Nanchang University, Nanchang, China

**Keywords:** low grade glioma, integrin subunit alpha V, prognosis, biomarker, biological function

## Abstract

As a member of integrin receptor family, ITGAV (integrin subunit α V) is involved in a variety of cell biological processes and overexpressed in various cancers, which may be a potential prognostic factor. However, its prognostic value and potential function in lower-grade glioma (LGG) are still unclear, and in terms of immune infiltration, it has not been fully elucidated. Here, the expression preference, prognostic value, and clinical traits of ITGAV were investigated using The Cancer Genome Atlas database (*n* = 528) and the Chinese Glioma Genome Atlas dataset (*n* = 458). Gene Ontology (GO) and Kyoto Encyclopedia of Genes and Genomes (KEGG) analyses and gene set enrichment analysis (GSEA) were used to explore the biological function of ITGAV. Using R package “ssGSEA” analysis, it was found thatthe ITGAV mRNA expression level showed intense correlation with tumor immunity, such as tumor-infiltrating immune cells and multiple immune-related genes. In addition, ITGAV is associated with some immune checkpoints and immune checkpoint blockade (ICB) and response to chemotherapy. and the expression of ITGAV protein in LGG patients was verified *via* immunohistochemistry (IHC). ITGAV expression was higher in LGG tissues than in normal tissues (*P* < 0.001) and multifactor analysis showed that ITGAV mRNA expression was an independent prognostic factor for LGG overall survival (OS; hazard ratio = 2.113, 95% confidence interval = 1.393–3.204, *P* < 0.001). GSEA showed that ITGAV expression was correlated with Inflammatory response, complement response, *KRAS* signal, and interferon response. ssGSEA results showed a positive correlation between ITGAV expression and Th2 cell infiltration level. ITGAV mRNA was overexpressed in LGG, and high ITGAV mRNA levels were found to be associated with poor protein expression and poor OS. ITGAV is therefore a potential biomarker for the diagnosis and prognosis of LGG and may be a potential immunotherapy target.

## Introduction

Glioma is one of the most common primary tumors of the central nervous system, accounting for approximately half of all primary intracranial space-occupying tumors (1). The annual incidences of glioma in my country amount to 3–8/100,000 people (2). Gliomas are highly invasive and have high morbidity and mortality rates (3). According to the World Health Organization (WHO) classification system, gliomas are classified between grades one (the lowest degree of malignancy and the best prognosis) and four (the highest degree of malignancy and the worst prognosis) (4). Among them, high-grade glioblastoma multiforme (GBM) has a poor prognosis (5), whereas the prognosis of low-grade glioma (LGG) is relatively good (6). However, some LGG patients have been found to have poor prognoses, which may be related to the heterogeneity of LGG; patients with isocitrate dehydrogenase (IDH) wild-type LGGs with estimated glomerular filtration rate amplification or with H3F3A mutations have been shown to exhibit worse clinical prognoses (7). At present, the main treatments for glioma include surgery, radiotherapy, chemotherapy, and targeted therapy (6, 8). The extent of tumor resection has a considerable impact on the median survival of patients (9). For patients with glioma, In clinical practice, patients often come to the hospital for treatment and diagnosis when they have symptoms in the late stage. Brain magnetic resonance imaging is the primary method for diagnosing early LGG (10), and early diagnosis and prompt treatment are important because they slow progression, delay the onset of new symptoms, and lead to a better prognosis. Therefore, there is an urgent need to identify valid and reliable biomarkers to determine poor prognosis and direct treatment strategies.

The integrin receptor family comprise the main receptors for cell adhesion and migration (11), cytoskeleton organization (12), cell proliferation (13), survival, and differentiation mediated by the extracellular matrix (14). The α-v integrin (ITGAV) consists of a subset that shares a common α-v subunit. ITGAV always binds to five β subunits to form receptors for hyaline, cellular actin, fibronectin, fibrinogen, and laminin (15). ITGAV is involved in many developmental processes. Integrin is highly expressed on the surfaces of a variety of tumors, and in neovascular endothelial cells (16); it also participates in the transmission process of calreticulin (CRT) on cell surfaces (17). Related studies have found that ITGAV signaling inhibits tumor cell apoptosis (18). In other diseases such as liver cancer, ITGAV has been shown to be involved in the occurrence and development of breast (19), nasopharyngeal (20), liver (21) and colorectal cancers (22). At the same time, it can bind to proteins on the cell surface (23) and regulate B cell signaling (24), which in turn plays an important role in tumors. However, few reports of ITGAV have been detailed regarding gliomas, and the expression pattern and prognostic value of ITGAV are still unclear; thus, this subject is worthy of further study.

Here, ITGAV was investigated as a novel prognostic factor for LGG. The expression preference, prognostic value, and biological function of ITGAV were evaluated using The Cancer Genome Atlas (TCGA) database. In addition, the prognostic value of ITGAV mRNA expression in LGG was validated using the Chinese Glioma Genome Atlas (CGGA) dataset. Next, Gene ontology (GO) and the Kyoto Encyclopedia of Genes and Genomes (KEGG) analyses and gene set enrichment analysis (GSEA) were performed to further understand the biological role of ITGAV in the pathogenesis of LGG. The correlation between ITGAV and tumor immune cell infiltration, immune checkpoint, immune-related genes, ICB and drug response was also examined. The expression level of ITGAV in tumors and its effect in glioma cell lines were verified by experiments. The expression of ITGAV protein in LGG patients was verified by immunohistochemistry (IHC), and a clinical model was developed to predict overall survival (OS) over different years. In short, ITGAV may be a new target for immunotherapy in the future.

## Materials and methods

### Datasets

This research includes from TCGA database (https://www.cancer.gov/about-nci/organization/ccg/research//structuralgenomicsTCGA) of two groups of data, RNA-seq transcriptome data and corresponding patient clinical data from LGG samples. From the TCGA database (https://portal.gdc.cancer.gov) to download the RNA sequence data of 528 patients with LGG, RNA-seq data and patient clinical information (workflow type: HTSEQ-FPKM) are captured using a data transfer tool (provided by GDC Apps) prognostic data were supplemented from a CELL article (25) and patient clinical characteristics were supplemented from Ceccarelli (26). Subsequent data processing excluded cases without survival data (*n* = 527). Patients with LGG were classified into low- and high-expression groups according to their median expression value of ITGAV. In this study, expression data and clinical data of LGG patients were also downloaded from the CGGA (http://www.cgga.org.cn/) database for external validation of survival analysis (27, 28), Subsequent data were extracted from patients with LGG (*n* = 458).

### Comparison of ITGAV expression levels between cancer tissue and corresponding normal tissue

The University of California Santa Cruz (UCSC) XENA (https://xenabrowser.net/datapages/) was used to download and extract TCGA LGG and corresponding normal tissue GTEx data. The expression levels of ITGAV in the tumor and normal groups were compared using amount of variance analysis. Differential expression analysis of ITGAV between glioma and normal brain tissues was performed using the R language package “ggplot2.”

### Survival analysis of ITGAV in LGG

To further verify the prognostic value of ITGAV mRNA expression in LGG, clinical data and expression matrix information from LGG patients with WHO grades of I-III in CGGA and TCGA were used. Similarly, the median value of ITGAV expression was used to divide these data into two groups: high expression and low expression. Kaplan–Meier survival analysis and the Cox proportional hazard model were used to estimate the prognostic value of ITGAV, based on TCGA and CGGA datasets using the R language packages “survival” and “survminer.”

### Analysis of differentially expressed genes (DEGs) between high and low ITGAV expression groups of LGG patients

Expression profiles (HTSeq-FPKM) were compared between the high and low ITGAV mRNA expression groups to identify DEGs using the unpaired Student's *t*-test; analysis was conducted using the R package “limma.” A |log2Fold Change| > 1.5 and adjusted *P* < 0.05 were considered as the threshold for DEGs.

### GO and KEGG enrichment analyses

Functional enrichment analyses, including GO analysis comprising cellular component (CC), molecular function (MF), and biological process (BP), and KEGG pathway analysis, were performed using the “clusterProfiler” package in R. Enriched ontological terms with an adjusted *P*-value of < 0.05 were regarded as being statistically significant.

### GSEA

GSEA is an analytical method that can determine whether a previously defined genome has a statistically significant and consistent difference between the two phenotypes. In this study, GSEA was performed using the R package “clusterProfiler” (29) to clarify the significant functional and pathway differences between the high ITGAV and low ITGAV groups. Gene set alignment was performed 1,000 times, and the expression level of ITGAV mRNA was used as a phenotypic marker. In this study, h.all.v7.0.symbols.gmt (Hallmarks) was selected as the reference gene set in the MSigDB collection. Adjust *P* < 0.05, a false discovery rate (FDR) of < 0.25, and a standardized enrichment score (|NES|)>1 was regarded to imply significant enrichment. Analysis of Connection Between ITGAV Expression Level and Immune Infiltration.Using the R tool “ssGSEA” (single sample gene set enrichment analysis) within the “GSVA” package (30, 31), the relative tumor infiltration levels of immune cell types were determined by integrating the gene expression levels in the published signature gene list. Wilcoxon rank sum and Pearson's correlation tests were performed to evaluate the relationship between immune cell infiltration and the different ITGAV mRNA expression groups.

### Prognostic model generation and prediction

Univariate and multivariate analyses were performed using Cox proportional risk models to estimate mortality risk, where *P* < 0.05 was considered statistically significant. The WHO grade, 1P/19q codeletion, IDH status, and Age variables were included in the construction of a clinical risk profile column diagram that was used to predict OS incidences at 1, 2, and 3 yr.

### Statistical analysis

Kruskal-Wallis, Wilcoxon signature, and Chi-square tests were used to analyze the relationship between clinicopathological features and ITGAV expression. The survival curve was drawn using the Kaplan-Meier method, and the difference between groups was evaluated using the logarithmic rank test. Univariate and multivariate analyses were performed using Cox proportional risk models to estimate mortality risk. In the multivariate Cox regression, there were 527 original data, 63 samples with missing variable information, and 464 samples were finally included. A value of *P* < 0.05 was considered to represent statistical significance.

### Association between ITGAV and immune-related genes, immune checkpoints, and immune checkpoint blockade responses

Using the R packages Limma, Reshape2, RColorBrewer, GGPLOT2, PheATMap and ImmuneeconV were used to analyze the correlation between ITGAV and immune-related genes or immune checkpoints. Using the TIDE algorithm to predict the potential response of the ICB, according to the existing pharmacogenomics Cancer Drug Sensitivity database (https://www.cancerrxgene.org/), use the ridge regression method to predict the sample 50% inhibitory concentration (IC50), the largest The R package method is used to predict the prediction accuracy.

### RNA isolation and quantitative reverse transcription-polymerase chain reaction (qRT-PCR)

qRT-PCR was used to compare with expression level of ITGAV mRNA in normal brain tissue (*n* = 6) and tumor tissue (*n* = 13), The 19 tissue samples were provided by Jiangxi Key Laboratory of Translational Cancer Research. In brief, according to the manufacturer's instructions, total RNA was extracted from LGG and normal samples using the TRIzol reagent (Invitrogen, Carlsbad, CA, USA). Then, complementary deoxyribonucleic acid (cDNA) was synthesized from 1 μg of total RNA using the Primrip TMRT reagent kit (Takara, Japan). Finally, the expression of ITGAV mRNA was detected by qRT-PCR using Eraser™ (Takara, Dalian, China), with glyceraldehyde 3-phosphate dehydrogenase (GAPDH) as the normalized control. The primers were as follows: ITGAV: F:5′-3′ GCTGTCGGAGATTTCAATGGT, R:5′-3′ TCTGCTCGCCAGTAAAATTGT.GAPDH: F:5′-CCCATCACCATCTTCCAGGAG-3′, R:5′-GTTGTCATGGATGACCTTGGC-3′.

### Cell culture

U251 and HS683 cell lines were obtained from the American culture collection (Manassas, Virginia, USA). Cells were grown in high glucose DMEM medium supplemented with 10% fetal bovine serum, penicillin-streptomycin mix, and 2 mM glutamine (all from Gibco/Invitrogen Technologies) at 37°C in a humidifified incubator with 5% CO2.Cell viability was determined by Cell Titer 96 Aqueous Reagent (MTS) colorimetric assay (Promega, Madison, WI, USA). In brief, cells (2 × 103 cells per well) were inoculated in 96-well plates 24 h before the experiment. The cells were then divided into three groups (NC, si-ITGAV) and incubated for 0, 24, 48, 72, or 96 h. Then, 10 μl of CellTiter 96 Aqueous One Solution Reagent was added to each well and incubated at 37°C for 30 min after the specifified time. Absorbance was then measured at 490 nm using a microplate reader (Bio-Rad Laboratories, Inc., Hercules, CA, USA). All experiments were conducted in triplicate.

### IHC

This study was approved by the Review Committee of the Second Affiliated Hospital of Nanchang University in Jiangxi Province, China. Tissue samples were obtained from 30 patients with pathological LGG and 6 patients with epilepsy who were treated at the Second Affiliated Hospital of Nanchang University in Jiangxi Province from 2019 to 2021 to detect ITGAV expression using IHC method. The 5-μm-thick glioma tissue sections were dewaxed in xylene, rehydrated through decreasing concentrations of ethanol and washed in distilled water. According to the standard protocols, sections were processed and stained with hematoxylin and eosin (H&E) and diaminobezidine. At last, sections were dehydrated through increasing concentrations of ethanol and xylene to the transparent state, and sealed with neutral gum. Follow the manufacturer's instructions to select the most suitable dilution concentration of 1.5:500. ITGAV positive cells were observed under ×400 and ×200 microscopies and ICH results were evaluated by two pathologists respectively.

## Results

### Upgrading of ITGAV in LGG

**Figure 1:**
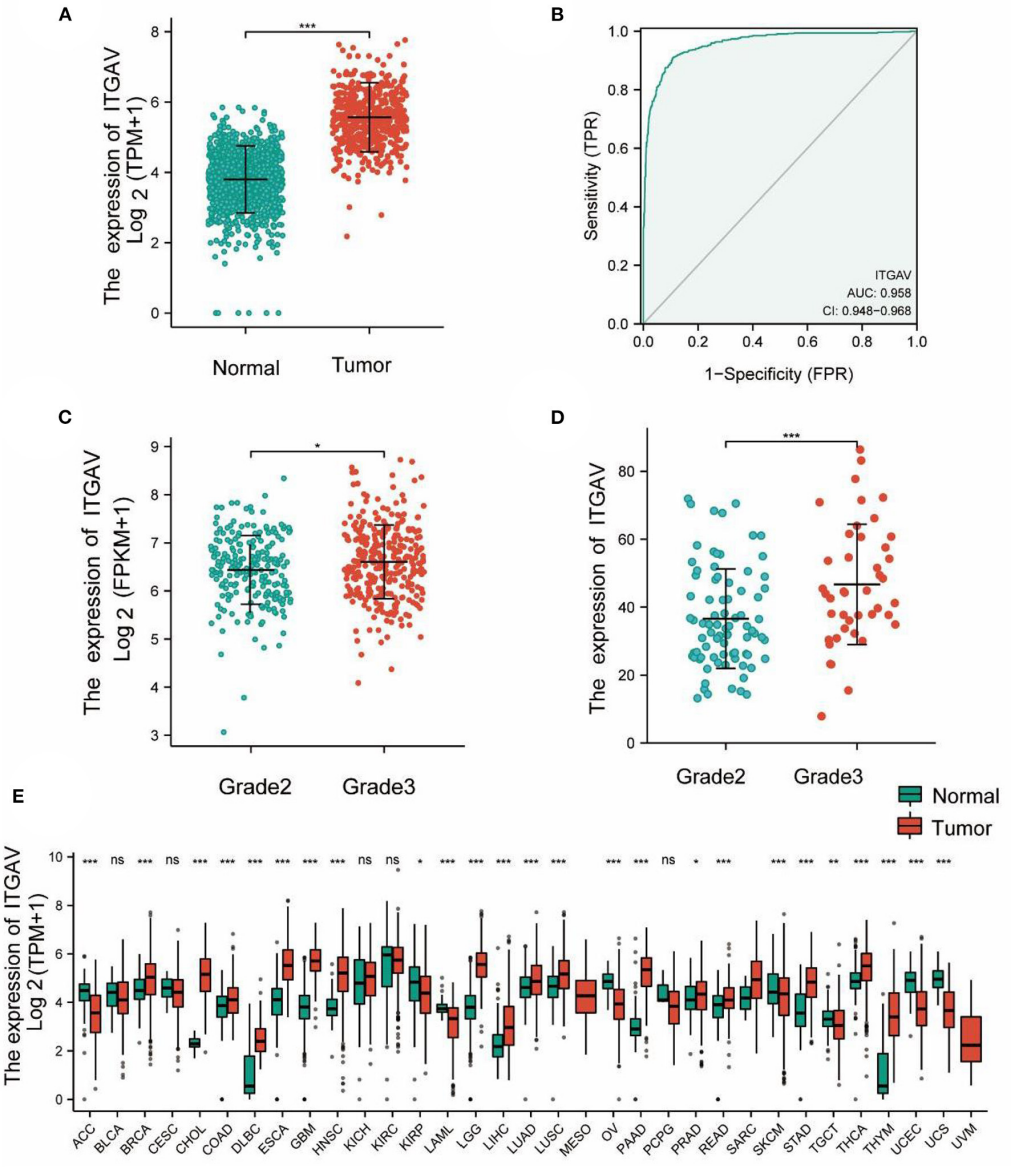
ITGAV mRNA in LGG and other types of human cancers from TCGA and CGGA data. **(A)** Expression levels of ITGAV in LGG and normal tissue. **(B)** Receiver operating characteristic analysis (ROC) of ITGAV in LGG. **(C)** The association of ITGAV expression and clinical Grade in LGG from TCGA data. **(D)** The association of ITGAV expression and clinical Grade in LGG from CGGA data. **(E)** ITGAV expression levels in different tumor types from TCGA database.

The results showed that the expression of ITGAV in LGG tissues was higher than that in normal tissues (*P* < 0.001; Figure 1A). The expression of ITGAV mRNA in the LGG group was signally higher than that in adjacent normal tissues. As can be seen from the receiver operating characteristic (ROC) results, the expression level of ITGAV mRNA in LGG was 0.958 (95% confidence interval (CI) = 0.948-0.968; Figure 1B); the optimal critical value of ITGAV was 4.600. At the same time, the expression of ITGAV mRNA was found to increase with increasing tumor invasiveness in gliomas of different grades (*P* < 0.05; Figures 1C,D). Compared to normal tissue, for approximately all tumor types in TCGA database, ITGAV mRNA expression was significantly overexpressed in invasive breast carcinoma (BRCA), colonic adenocarcinoma (COAD), cholangiocarcinoma (CHOL), esophageal carcinoma (ESCA), GBM, head and neck squamous cell carcinoma (HNSC), kidney chromophobe (KICH), hepatocellular carcinoma (LIHC), lung adenocarcinoma (LUAD), lung squamous cell carcinoma (LUSC), pheochromocytoma, paraganglioma (PCPG), rectal adenocarcinoma (READ), gastric adenocarcinoma (STAD), thymic carcinoma, and prostate carcinoma (PRAD; Figure 1E).

### MRNA expression levels of ITGAV in LGG

**Figure 2:**
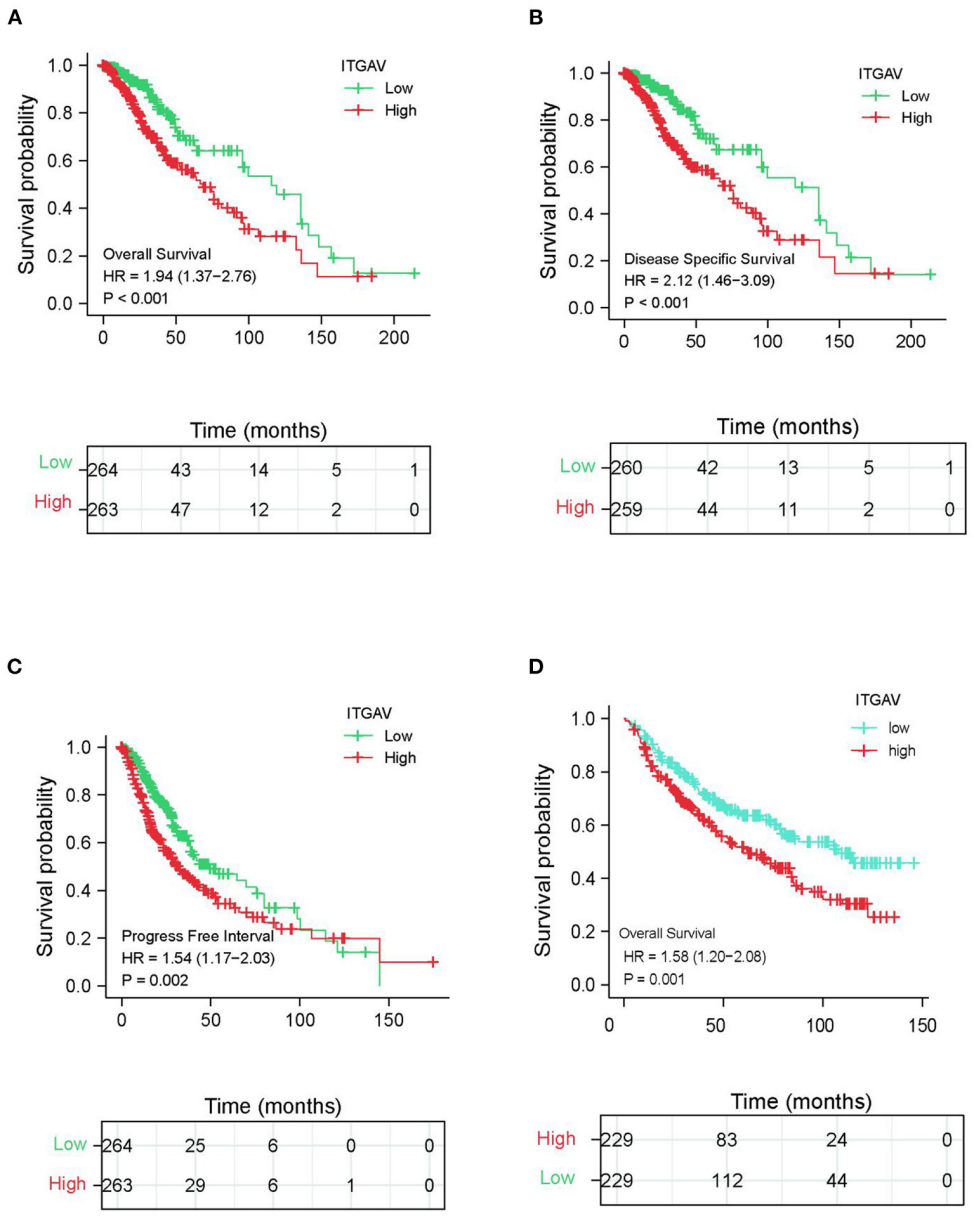
The prognostic value of ITGAV expression in LGG. **(A)** Survival curves of OS from TCGA data (*n* = 527). **(B)** Survival curves of DSS from TCGA data (*n* = 519). **(C)** Survival curves of PFI from TCGA data (*n* = 527); **(D)** Survival curves of OS from CGGA data (*n* = 458).

The total sample size was 528, including 239 male and 289 female patients. Subsequent data processing excluded cases without survival data (*n* = 527). Due to the lack of clinical sample information, the number of the univariate and multivariate analyses was 464. According to the median expression level of ITGAV in LGGs, the total samples were divided into low-expression and high-expression groups. Detailed clinicopathological features are shown in Table 1. Kaplan-Meier survival analysis showed that those patients with high ITGAV expressions in TCGA-LGG dataset had poorer prognoses and lower OS (hazard rate (HR) = 1.94, 95% CI = 1.37–2.76, *P* < 0.001; Figure 2A). Multivariate analysis also showed that ITGAV mRNA expression was an independent prognostic factor of OS for LGG (HR = 2.113, 95% CI = 1.393-3.204, *P* < 0.001). In addition, WHO grade (HR = 2.215, 95% CI = 1.434-3.420, *P* < 0.001), 1p/19q codeletion (HR = 1.806, 95% CI = 1.075–3.035, *P* < 0.001), IDH status (HR = 0.275, 95% CI = 0.172–0.439, *P* < 0.001), and Age (HR = 2.681, 95% CI = 1.735–4.143, *P* < 0.001) were also independent prognostic factors (Table 2). Kaplan-Meier analysis showed that the disease-specific survival rate of TCGA-LGG group with high ITGAV mRNA expressions was lower than that of the low expression group (HR = 2.12, 95% CI = 1.48–3.09, *P* < 0.001; Figure 2B). Kaplan-Meier analysis showed that the progression-free survival (PFS) rate of TCGA-LGG group with high ITGAV mRNA expressions was lower than that of the low expression group (HR = 1.54, 95% CI = 1.17–2.03, *P* = 0.002; Figure 2C). Kalan-Meier analysis showed that OS for patients with high ITGAV was poor (HR = 1.58, 95% CI = 1.20–2.08, *P* = 0.001; Figure 2D). Multivariate analysis conducted using CGGA data showed that ITGAV mRNA was also an independent prognostic factor for LGG (HR = 1.282, 95% CI = 1.089–1.510, *P* = 0.003). In addition, WHO grade (HR = 2.601, 95% CI = 1.879–3.600, *P* < 0.001), IDH mutation status (HR = 0.605, 95% CI = 0.447–0.819, *P* = 0.001), and 1p/19q codeletion status (HR = 0.343, 95% CI = 0.232–0.506, *P* < 0.001) were also independent prognostic factors (Table 3).

**Table 1:** Demographic and clinical characteristics of LGG patients with low and high expression ITGAV in TCGA (*n* = 528).

**Characteristic**	**Low expression of ITGAV**	**High expression of ITGAV**	**p**
n	264	264	
WHO grade, *n (%)*			0.334
G2	117 (25.1%)	107 (22.9%)	
G3	115 (24.6%)	128 (27.4%)	
IDH status, *n (%)*			< 0.001
WT	31 (5.9%)	66 (12.6%)	
Mut	232 (44.2%)	196 (37.3%)	
1p/19q codeletion, *n (%)*			0.009
codel	100 (18.9%)	71 (13.4%)	
non–codel	164 (31.1%)	193 (36.6%)	
Gender, *n (%)*			0.484
Female	115 (21.8%)	124 (23.5%)	
Male	149 (28.2%)	140 (26.5%)	
Age, *n (%)*			0.663
< =40	135 (25.6%)	129 (24.4%)	
>40	129 (24.4%)	135 (25.6%)	
Race, *n (%)*			0.091
Asian	6 (1.2%)	2 (0.4%)	
Black or African American	7 (1.4%)	15 (2.9%)	
White	246 (47.6%)	241 (46.6%)	
OS event, *n (%)*			< 0.001
Alive	215 (40.7%)	177 (33.5%)	
Dead	49 (9.3%)	87 (16.5%)	
DSS event, *n (%)*			< 0.001
Alive	218 (41.9%)	179 (34.4%)	
Dead	42 (8.1%)	81 (15.6%)	
PFI event, *n (%)*			0.002
Alive	177 (33.5%)	141 (26.7%)	
Dead	87 (16.5%)	123 (23.3%)	
Age, meidan (IQR)	40 (32, 52)	41 (32, 53)	0.370

**Table 2:** The univariate and multivariate analyses of overall survival according to ITGAV expression, after adjusting for other potential predictors in TCGA (*n* = 464).

**Characteristics**	**Univariate analysis**		**Multivariate analysis**	

	**Hazard ratio (95% CI)**	***P*** **–value**	**Hazard ratio (95% CI)**	***P*** **–value**
WHO grade (G3 vs. G2)	3.059 (2.046–4.573)	**<0.001**	2.215 (1.434–3.420)	**<0.001**
1p/19q codeletion (non–codel vs codel)	2.493 (1.590–3.910)	**<0.001**	1.806 (1.075–3.035)	**0.025**
IDH status (Mut vs. WT)	0.186 (0.130–0.265)	**<0.001**	0.275 (0.172–0.439)	**<0.001**
Gender (Male vs. Female)	1.124 (0.800–1.580)	0.499		
Age (>40 vs. < =40)	2.889 (2.009–4.155)	**<0.001**	2.681 (1.735–4.143)	**<0.001**
Laterality (Left & Midline vs. Right)	1.298 (0.921–1.831)	0.137		
ITGAV (High vs. Low)	1.859 (1.310–2.639)	**<0.001**	2.113 (1.393–3.204)	**<0.001**

The bold values provided represent *p* < 0.05.

**Table 3:** The univariate and multivariate analyses of overall survival according to ITGAV expression, after adjusting for other potential predictors in CGGA (*n* = 458).

**Characteristics**	**Univariate analysis**		**Multivariate analysis**	

	**Hazard ratio (95% CI)**	***P*** **–value**	**Hazard ratio (95% CI)**	***P*** **–value**
WHO grade	2.984 (2.214–4.023)	**<0.001**	2.601 (1.879–3.600)	**<0.001**
(G2 vs G3)				
Gender (Male vs Female)	0.973 (0.738–1.283)	0.848		
Age (>40 vs. < =40)	1.232 (0.936–1.621)	0.136		

The bold values provided represent *p* < 0.05.

### Prognostic potential of ITGAV in human LGG cases

**Figure 3:**
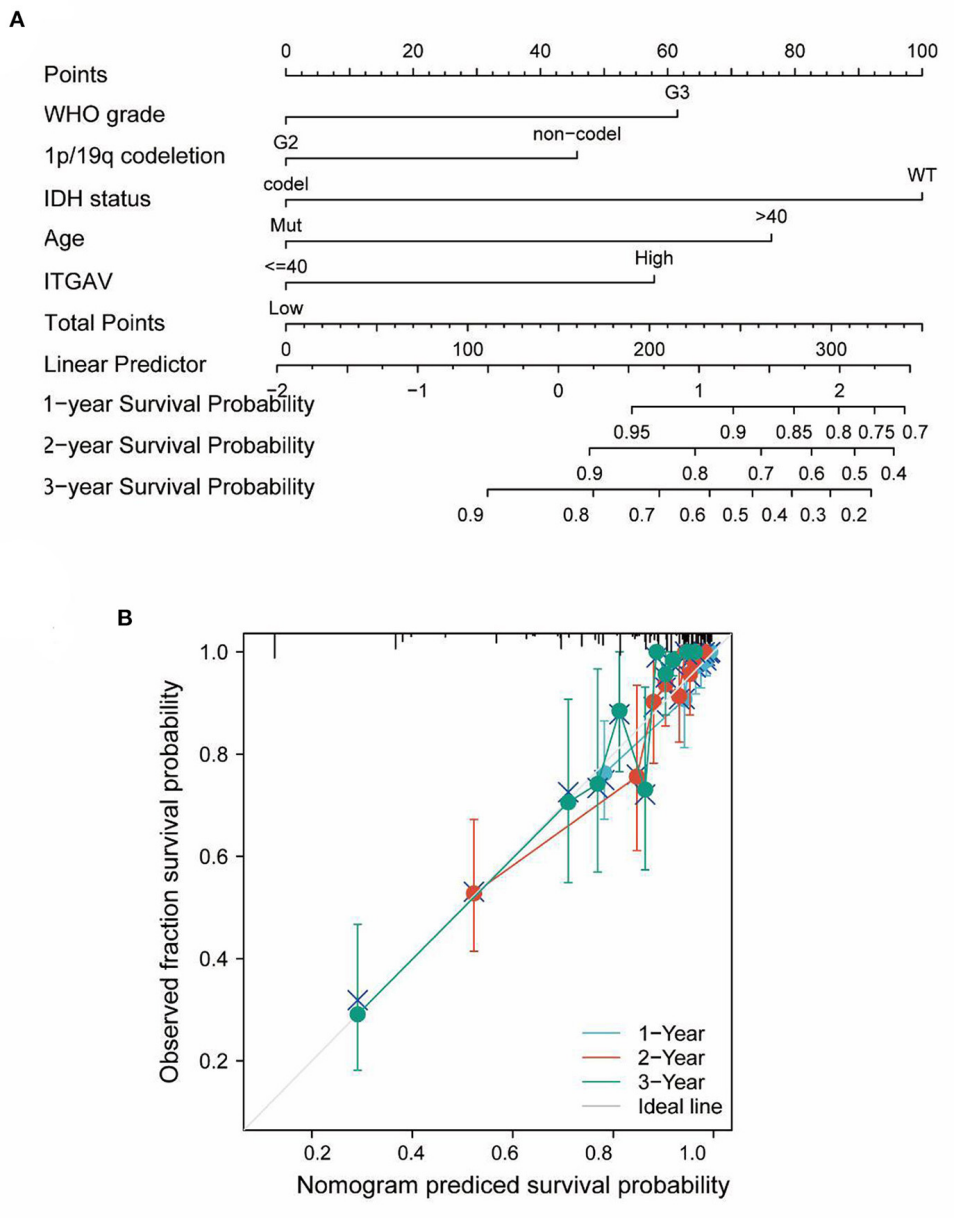
Prognostic Model Generation and Prediction with ITGAV. **(A)** A nomogram that integrates ITGAV and other prognostic factors in LGG from TCGA data. **(B)** The calibration curve of the nomogram.

These results suggest that ITGAV mRNA is an independent prognostic factor for LGG. This inference was verified by fitting ITGAV mRNA expression and other clinicopathological parameters to establish predictive models of OS and PFS using TCGA data. An OS histogram was incorporated by incorporating ITGAV and prognostic factors including WHO grade, IDH mutation status, 1p/19q co-deletion status, and age (Figure 3A); the higher the point on the chart, the worse the prognostic factor. Calibration curves were used to evaluate the performances of the ITGAV diagrams, and the C-index of OS was 0.812 (0.790–0.835; Figure 3B). In summary, this column may be a better predictor of survival in LGG patients than any single prognostic factor.

### Functional enrichment analysis of high–and low-ITGAV expression samples

**Figure 4:**
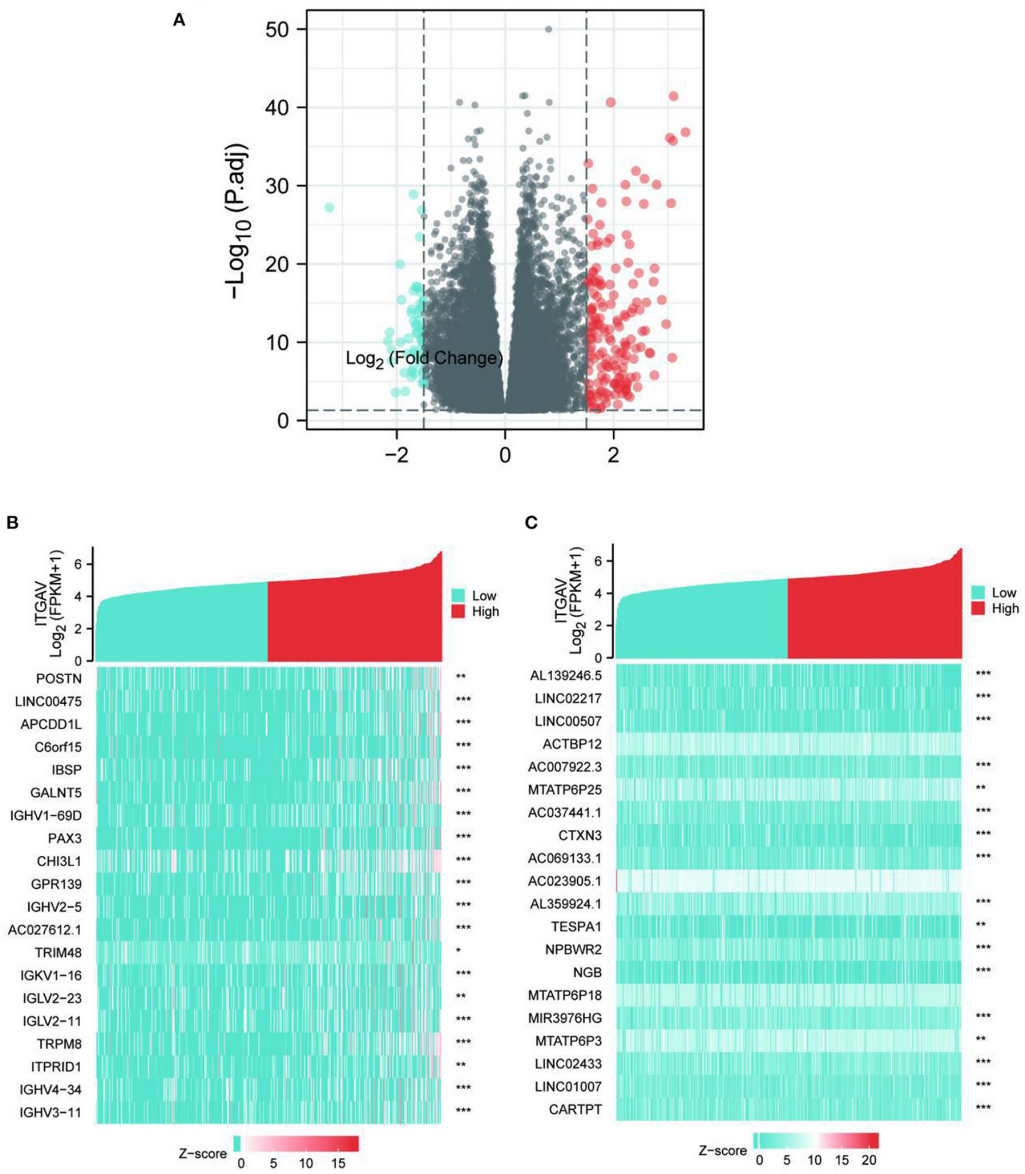
**(A)** Volcano maps of differentially expressed genes. **(B,C)** Heat maps of differentially expressed genes.

**Figure 5:**
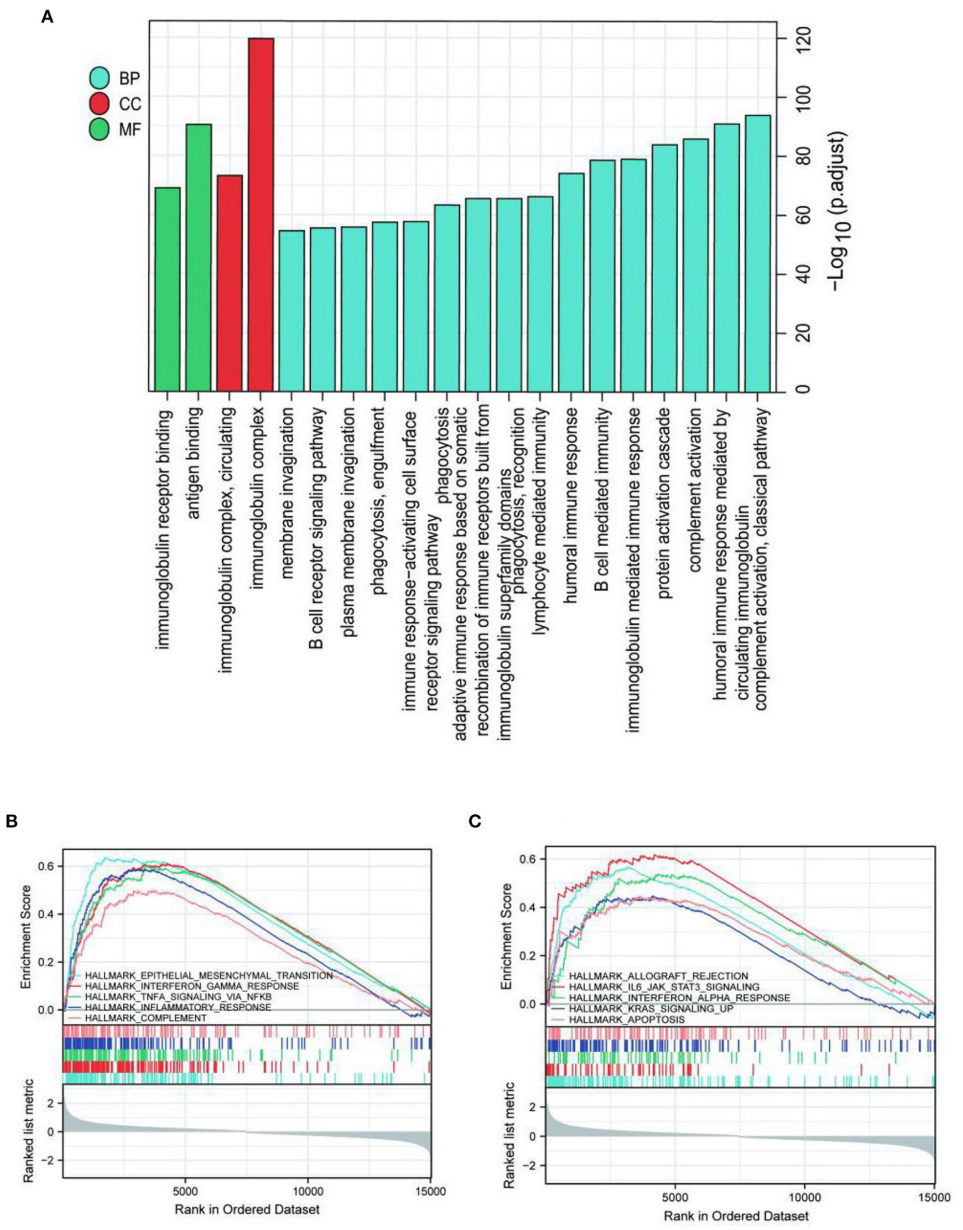
Functional enrichment of ITGAV in LGG. **(A)** GO and KEGG enrichment analysis of differentially expressed genes. **(B,C)** The most significant enrichment pathways by GSEA.

Limma was analyzed in high- and low-expression ITGAV samples to explore the potential mechanisms by which ITGAV promotes tumor progression. A total of 227 DEGs were identified, among which 169 were up-regulated and 58 were down-regulated (Figures 4A–C). GO enrichment and KEGG analysis revealed the Top go BP, MF, and CC groups, including immunoglobulin complex, complement activation, classical pathway, humoral immune response mediated by circulating immunoglobulin, antigen binding, protein activation cascade, immunoglobulin mediated immune response, B cell mediated immunity, humoral immune response, immunoglobulin receptor binding (Figure 5A). Furthermore, GSEA was used to identify key pathways associated with ITGAV, revealing that 22 datasets met the threshold of FDR < 0.25 and adjust *P* < 0.05, as shown in Table 4. The most significant enrichment pathways were Epithelial mesenchymal transtion, Interferon gamma response, TNFA signaling *via* NF-kB, Inflammatory response, Complement respond (Figures 5B,C).

**Table 4:** Hallmark pathways enriched in high– and low–risk groups by using GSEA.

**ID**	**NES**	***p*** **–value**	**P adjust**
HALLMARK_COMPLEMENT	2.536329165	0.001703578	0.006277464
HALLMARK_HYPOXIA	1.951312778	0.001715266	0.006277464
HALLMARK_IL2_STAT5_SIGNALING	2.282956153	0.001715266	0.006277464
HALLMARK_INFLAMMATORY_RESPONSE	3.00543963	0.001724138	0.006277464
HALLMARK_TNFA_SIGNALING_VIA_NFKB	3.070584872	0.001724138	0.006277464
HALLMARK_G2M_CHECKPOINT	1.958029265	0.001733102	0.006277464
HALLMARK_KRAS_SIGNALING_UP	2.257371199	0.001733102	0.006277464
HALLMARK_ALLOGRAFT_REJECTION	2.913928669	0.00174216	0.006277464
HALLMARK_EPITHELIAL_MESENCHYMAL_TRANSITION	3.291234464	0.00174216	0.006277464
HALLMARK_APOPTOSIS	2.163615571	0.001751313	0.006277464
HALLMARK_INTERFERON_GAMMA_RESPONSE	3.124229074	0.001757469	0.006277464
HALLMARK_COAGULATION	2.419716356	0.001769912	0.006277464
HALLMARK_ANGIOGENESIS	2.433944967	0.001865672	0.006277464
HALLMARK_INTERFERON_ALPHA_RESPONSE	2.382558229	0.001865672	0.006277464
HALLMARK_IL6_JAK_STAT3_SIGNALING	2.662199864	0.001883239	0.006277464
HALLMARK_OXIDATIVE_PHOSPHORYLATION	−1.875867136	0.002369668	0.007405213
HALLMARK_UV_RESPONSE_DN	1.967580111	0.003521127	0.010356255
HALLMARK_ANDROGEN_RESPONSE	1.670435043	0.005758157	0.015994882
HALLMARK_KRAS_SIGNALING_DN	−1.547793405	0.007058824	0.018575851
HALLMARK_E2F_TARGETS	1.577790982	0.010471204	0.02617801
HALLMARK_MITOTIC_SPINDLE	1.475771468	0.013937282	0.033184005
HALLMARK_PROTEIN_SECRETION	1.567622526	0.018348624	0.041701418

### ITGAV expression is correlated with immune infiltration levels in LGG

**Figure 6:**
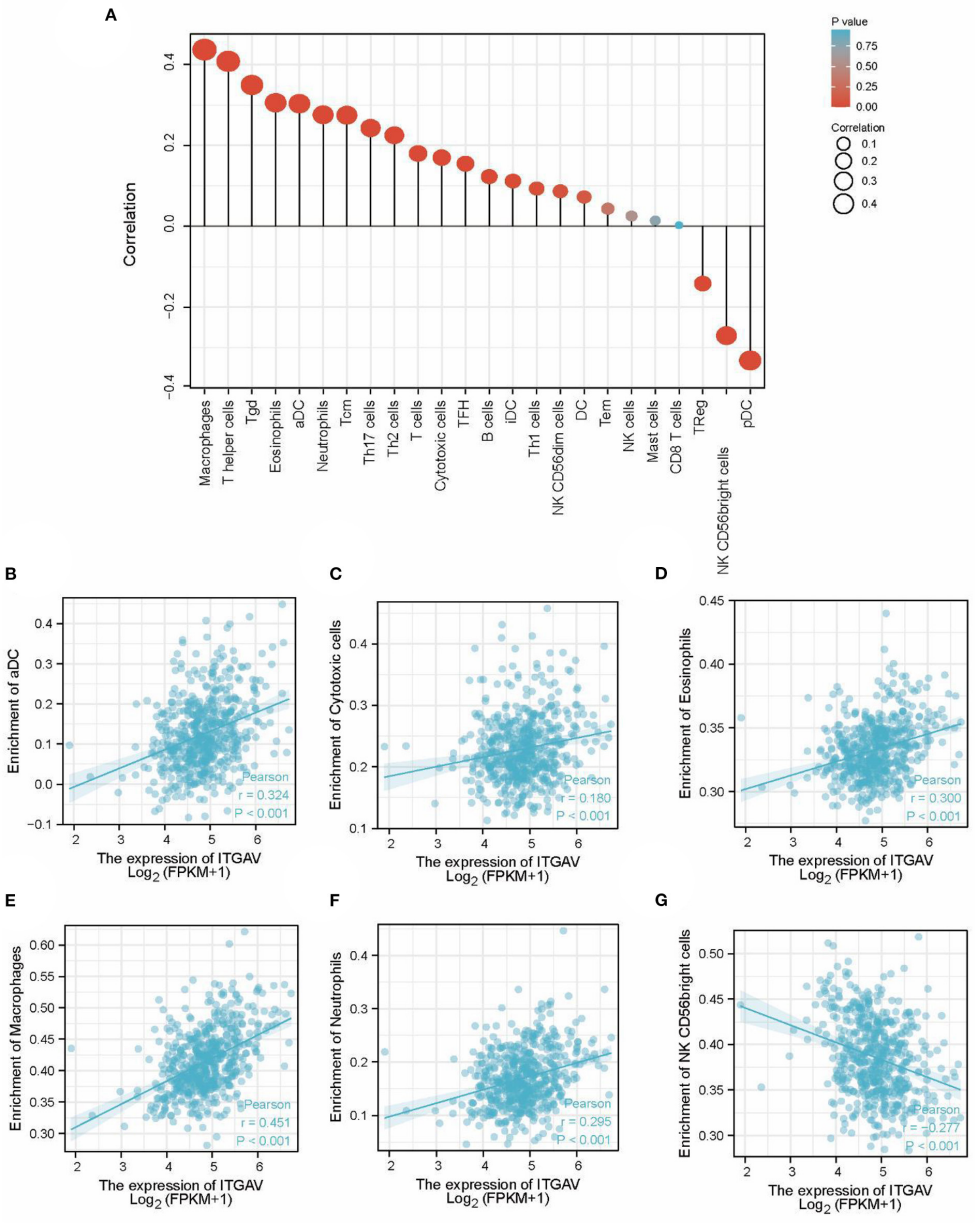
ssGSEA analyses of ITGAV and the correlation of ITGAV expression with immune infiltration level in LGG. **(A)** The correlation between the infiltration of immune cells and the expression of ITGAV. **(B–G)** ITGAV expression significantly positively correlates with infiltrating levels of aDC, Cytotoxic cells, Neutrophil, T cells, Eosinophils, and Macrophages.

Both KEGG and GSEA revealed that ITGAV may be involved in tumor immune response. Thus, ssGSEA was further used to analyze the relationship between ITGAV mRNA expression and the level of immune cell infiltration (Table 5); the correlation between the two is shown in Figure 6A. ITGAV mRNA expression was found to be correlated with activated dendritic cells(aDC) (R = 0.324, *P* < 0.001; Figure 6B), cytotoxic cells (R = 0.180, *P* < 0.001; Figure 6C), neutrophils (R = 0.295, *P* < 0.001; Figure 6D), T cells (R = 0.192, *P* < 0.001; Figure 6E), eosinophils (R = 0.300, *P* < 0.001; Figure 6F), macrophages (R = 0.451, *P* < 0.001; Figure 6G), T helper cells (R = 0.391, *P* < 0.001; Supplementary Figure 1A), T central memory(Tcm) (R = 0.271, *P* < 0.001; Supplementary Figure 1B), T follicular helper(TFH) (R = 0.202, *P* < 0.001; Supplementary Figure 1C), T gamma delta(Tgd) (R = 0.388, *P* < 0.001; Supplementary Figure 1D), Th17 cells (R = 0.206, *P* < 0.001; Supplementary Figure 1E), Th2 cells (R = 0.190, *P* < 0.001; Supplementary Figure 1F), B cells (R = 0.158, *P* < 0.001; Supplementary Figure 1G), immature dendritic cells(iDC) (R = 0.114, *P* = 0.009; Supplementary Figure 1H), Th1 cells (R = 0.163, *P* < 0.001; Supplementary Figure 1I), and natural killer (NK) CD56dim cells (R = 0.116, *P* = 0.007; Supplementary Figure 2A) were all positively correlated with infiltration. In addition, ssGSEA also showed that ITGAV expression was negatively correlated with NK CD56bright cells (R = −0.277, *P* < 0.001; Supplementary Figure 2B), plasmacytoid dendritic cells(pDC) (R = −0.315, *P* < 0.001; Supplementary Figure 2C), and regulatory T cells (TReg) (R = −0.114, *P* = 0.009; Supplementary Figure 2D). However, ITGAV mRNA expression was not significantly correlated with the infiltration of DC (R = 0.081, *P* = 0.063; Supplementary Figure 2E), T effector memory (Tem) (R = 0.065, *P* = 0.133; Supplementary Figure 2F), NK cells (R = 0.060, *P* = 0.167; Supplementary Figure 2G), mast cells (R = 0.028, *P* = 0.523; Supplementary Figure 2H), CD8 T cells (R = 0.006, *P* = 0.886; Supplementary Figure 2I).

**Table 5:** The relationship between ITGAV mRNA expression and the level of immune cell infiltration.

**ITGAV**	**Cells**	**Correlation** **(Pearson)**	***P*** **–value(Pearson)**	**Correlation** **(Spearman)**	***P*** **-value(Spearman)**
	aDC	0.324	<0.001	0.303	<0.001
	Cytotoxic cells	0.180	<0.001	0.170	<0.001
	Eosinophils	0.300	<0.001	0.306	<0.001
	Macrophages	0.451	<0.001	0.437	<0.001
	Neutrophils	0.295	<0.001	0.276	<0.001
	NK CD56 bright cells	−0.277	<0.001	−0.270	<0.001
	pDC	−0.315	<0.001	−0.332	<0.001
	T cells	0.192	<0.001	0.180	<0.001
	T helper cells	0.391	<0.001	0.408	<0.001
	Tcm	0.271	<0.001	0.275	<0.001
	TFH	0.202	<0.001	0.155	<0.001
	Tgd	0.388	<0.001	0.350	<0.001
	Th17 cells	0.206	<0.001	0.243	<0.001
	Th2 cells	0.190	<0.001	0.225	<0.001
	TReg	−0.114	0.009	−0.142	0.001
	B cells	0.158	<0.001	0.123	0.005
	iDC	0.114	0.009	0.112	0.010
	Th1 cells	0.163	<0.001	0.093	0.032
	NK CD56dim cells	0.116	0.007	0.087	0.046
	DC	0.081	0.063	0.072	0.096
	Tem	0.065	0.133	0.044	0.317
	NK cells	0.060	0.167	0.026	0.558
	Mast cells	0.028	0.523	0.014	0.746
	CD8 T cells	0.006	0.886	0.003	0.949

### Expression of ITGAV is related to immune checkpoints, immune checkpoint blockade(ICB), and sensitivity to chemotherapy

**Figure 7:**
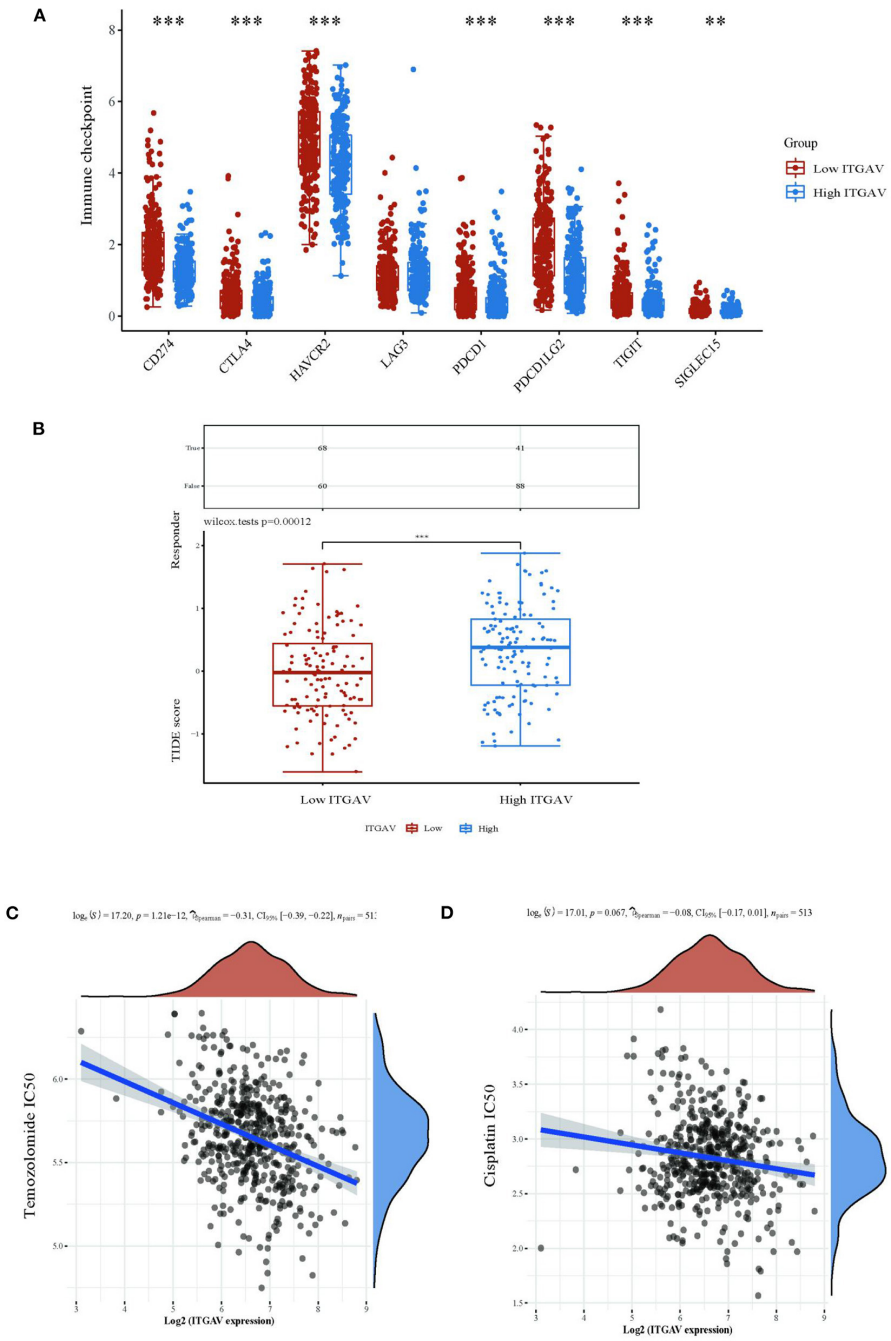
Expression of ITGAV Is related to immune checkpoints, immune checkpoint blockade(ICB), sensitivity to chemotherapy. **(A)** In LGG patients with high or low ITGAV expression level, the immune checkpoint expression is different between the two groups. **(B)** Different responses of ITGAV groups to immune checkpoint blockade in LGG patients. **(C,D)** Correlations between ITGAV and the IC50 of chemotherapy drugs. **p < 0.01, ***p < 0.001.

To further elucidate the role of ITGAV in immunity to lower grade gliomas, we also evaluated the differences in immune checkpoints between the two groups with high and low ITGAV expression levels. We found that CD274, CTLA4, HAVCR2, PDCD1, PDCD1LG2, TIGIT and SIGLEC15 were significantly different among different groups.With the exception of LGA3, ITGAV expression was visually positively correlated with immune checkpoint markers (Figure 7A). In addition, LGG patients with high ITGAV expression had a higher TIDE score, suggesting that these patients may respond better to ICB treatment (Figure 7B). We also evaluated the association between ITGAV and temozolomide and cisplatin, commonly used drugs for glioma. ITGAV expression was negatively correlated with the IC50 of temozolomide (Figures 7C,D).

### Knocking down ITGAV inhibited the proliferation and migration of glioma cells and validation of ITGAV expression and ITGAV prognostic value in LGG

**Figure 8:**
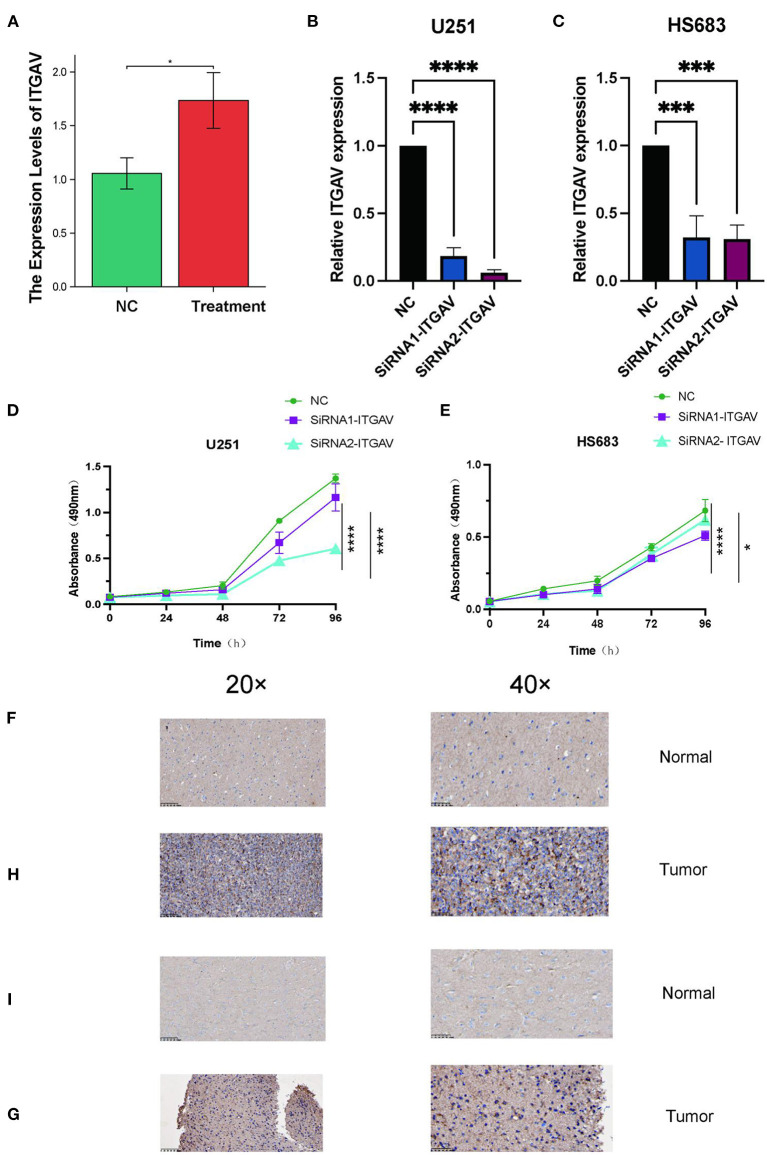
Knocking down ITGAV inhibited the proliferation and migration of glioma cells and validation of ITGAV expression and ITGAV prognostic value in LGG. **(A)** ITGAV mRNA expression in normal tissue (*n* = 6) and LGG tissues (*n* = 13). **(B,C)** In U251 and HS683 cells, the expression of ITGAV was downregulated in the siRNA group; **(D,E)** MTS assay to detec U251 transfected with si–ITGAV and the proliferation inhibition of HS683 cells. **(F)** The Expression of ITGAV in normal tissue (IHC). **(H)** The Expression of ITGAV in LGG tissue (IHC). **(I)** The Expression of ITGAV in normal tissue (IHC). **(G)** The Expression of ITGAV in LGG tissue (IHC).

To investigate the role of ITGAV in LGG, we evaluated the effect of ITGAV on lower–grade glioma cell proliferation. qRT–PCR analysis showed that compared with normal brain tissue the expression of ITGAV in LGG tissues was significantly higher than that in normal tissues (*p* < 0.05; Figure 8A); Four kinds of siRNA were used to inhibit ITGAV expression in U251 and HS683 cell lines. After transfection and incubation for 48 hrs, the interference efficiency of siRNA was detected by qRT–PCR. We found that it has high silencing efficiency (Figures 8B,C) MTS results showed that ITGAV knockdown reduced the number of U251 and HS683 cells compared with the NC group (Figures 8D,E). A total of 36 samples were used for immunohistochemistry, including 6 normal tissues and 30 tumor tissues, of which 6 were negative for ITGAV protein expression. Among the 30 tumor samples, 7 (23.3%) were negative and 23 (76.7%) were positive in immunohistochemistry.We performed IHC to test ITGAV protein expression in LGG tissues and their counterparts and to examine the expression of ITGAV in LGG. We found that ITGAV proteins were more highly expressed in the LGG tissues than in the normal tissues, In immunohistochemical staining, ITGAV protein was significantly positive in LGG patients (Figures 8F,G).

## Discussion

This study found that ITGAV was significantly overexpressed in LGG. Furthermore, high expression of ITGAV was found to be associated with poor prognosis, and functional enrichment analysis showed that ITGAV expression was related to *KRAS* signal transduction, immunoglobulin complex, complement activation, classical pathway, humoral immune response mediated by circulating immunoglobulin, antigen binding, protein activation cascade, immunoglobulin mediated immune response, B cell mediated immunity, humoral immune response, and immunoglobulin receptor binding, Interferon alpha response are all associated with increases in the levels of infiltration of various immune cells. This indicates that ITGAV expression may affect the immune microenvironment. We also found that ITGAV expression was positively correlated with most immune checkpoint markers, and immune checkpoint genes were exactly an important factor leading to tumor immune escape, which further confirmed the important role of ITGAV in immunotherapy. Moreover, LGG patients with high ITGAV expression had higher TIDE scores, suggesting that these patients may respond better to ICB treatment. Therefore, this study provides insights into the potential role of ITGAV in tumor pathogenesis and demonstrates its application prospects as a potential biomarker for LGG.

This study showed that, in both TCGA database and the presented specimen data (*P* < 0.001), ITGAV was highly expressed in LGG. These results are consistent with previous studies, which have also found that ITGAV proteins are highly expressed in various types of cancer, including pancreatic (32), liver (21), and colorectal cancers (33). The present analysis confirmed this, as it was found that ITGAV was significantly overexpressed in most tumors in TCGA data. These results suggest that ITGAV has the potential to be a diagnostic marker for a variety of cancers. In addition, ITGAV was also shown to be associated with the pathological type, IDH mutation, and 1P/19q codeletion status of LGG, further supporting the interpretation that ITGAV expression may be related to the malignant degree of LGG.

ITGAV is highly expressed in LGG and is associated with poor prognosis. Here, according to TCGA LGG data, patients with high expressions of ITGAV mRNA had poor OS; thus, it is an independent prognostic factor for OS and PFS. This result was verified in LGG data from the CGGA dataset, and multivariable analysis also revealed that ITGAV was an independent prognostic factor for LGG. In addition, this study suggests that ITGAV protein expression is a prognostic biomarker for LGG. A large number of related studies have shown that ITGAV expression may be a biomarker for poor prognoses regarding various tumors (19–22). Considering that ITGAV is a strong prognostic factor, here ITGAV expression was combined with clinical data to construct a graph that could predict the OS of patients with ITGAV at 1, 2, and 3 yr. This chart could help to screen high–risk patients and identify more aggressive treatment options for high–risk LGG patients.

As an integrin receptor, ITGAV plays roles in a variety of biological functions that lead to tumor development (34). Enrichment analysis revealed that ITGAV may be involved in the *KARS* signaling pathway, androgen response, protein secretion, and other pathways in LGG. Previous studies have found that ITGAV may be involved in a variety of biological functions, such as cell proliferation, cycle regulation, migration, and invasion. Interestingly, GSEA revealed that ITGAV may be involved in inflammation, and may also complement pathways. In addition, ssGSEA also showed that ITGAV was positively correlated with Th2 cell infiltration, but not with the infiltration of CD8 T cells, Mast cells, NK cells, or DC cells. Previous studies have found that Th2 cell infiltration was associated with Th2 cell immunosuppression and poor survival regarding various tumors (35–37). In this study, a significant increase was observed in Th2 cells, suggesting that ITGAV may be involved in LGG–mediated immune escape. A similar situation has also been reported for the tumor–associated antigen *EpCAM*, which promotes Th2 cell–mediated immune escape (37). In our study, we found that CD274, CTLA4, HAVCR2, PDCD1, PDCD1LG2, TIGIT and SIGLEC15 increased with the increase of ITGAV expression level. Previous studies have shown that these immune checkpoint molecules are expressed on immune cells, resulting in immune escape from tumor formation. This explains the important role of ITGAV in tumor immunity. Interestingly, ITGAV was also inversely correlated with the semi–inhibitory concentration of temozolomide, a commonly used chemotherapy drug. This suggests that patients with lower grade gliomas with high ITGAV expression may benefit better from ICB treatment. These results suggest that ITGAV may be a predictor of ICB and chemotherapy. These findings provide new insights into the precise targeting of treatment in patients with LGG.

Here, ITGAV expression was found to be increased in LGG and high expression was shown to be a factor in poor prognosis. This suggests that ITGAV may be a therapeutic target for LGG. CARs are synthetic receptors that redirect T cells to tumor surface antigens (38). As a popular immunotherapy method (39), the application value of CARs in primary brain tumors and metastatic brain tumors is worth exploring. Previous studies have speculated that CAR T may improve treatment outcomes in patients with Glioblastoma (40). Given that ITGAV can be expressed on tumor surfaces (41), ITGAV is may be a potential target for CAR–T therapy, but future research is needed, Therefore, ITGAV is of great value as a future possible immunotherapy target.

Though this study achieved a deeper understanding of the relationship between ITGAV and LGG, there are also some limitations. First, the lack of *in vitro* and *in vivo* experimentations made it impossible to validate the obtained results. In addition, due to the design limitations of the study, other key signaling pathways associated with ITGAV may have been missed; this issue require furthers study. Before the study, ITGAV expression levels were grouped and KM curves were verified in GBM, and the results showed that there was no difference between the two groups. This may be related to the small sample size of glioblastoma in TCGA. Therefore, from the clinical significance, ITGAV may be more instructive for the prognosis of lower–grade glioma. So we didn't include glioblastoma in the study. Recent C–ImpacT–NOW updates 3 and 5, which discuss emerging data, point to the importance of molecular characteristics of gliomas and make us realize that histological grades are not sufficient to describe gliomas. It is clear from our analysis that increased ITGAV expression is associated with poorer survival, and that these increased levels are more common in IDH wild–type, non-coding deletion, and or G3 tumors. However, from a clinical point of view, IDH wild G2 glioma is considered invasive and is considered an aggressive high–grade glioma. This needs our further analysis and exploration.

## Conclusion

ITGAV mRNA was shown to be overexpressed in LGG, and high ITGAV mRNA was found to be associated with poor protein expression and OS. ITGAV is a potential biomarker for the diagnosis and prognosis of LGG and may be a potential target for immunotherapy.

## Data availability statement

The original contributions presented in the study are included in the article/Supplementary material, further inquiries can be directed to the corresponding author.

## Ethics statement

Written informed consent was obtained from the individual(s) for the publication of any potentially identifiable images or data included in this article.

## Author contributions

ZT and X-LS conceived and designed the experiments. ZZ, KY, HY, and HL collected the data. ZT and ZZ analyzed the data. ZT and YJ wrote the article. TL, JC, WH, ZC, and YM conducted quality control on the articles and guided the submission. All authors read and approved the final manuscript.

## Funding

This study was supported by the National Natural Science fund project (81960458), The Province Natural Science Foundation of Jiangxi Province (No. 20192BAB215063), The research program of science and technology in Jiangxi province department of education (180077), Youth Project of Jiangxi Provincial Department of Education (GJJ200244), Science and Technology Program of Jiangxi Provincial Health Commission (202130347), and Project of the Second Affiliated Hospital of Nanchang University (2014YNLC12009).

## Conflict of interest

The authors declare that the research was conducted in the absence of any commercial or financial relationships that could be construed as a potential conflict of interest.

## Publisher's note

All claims expressed in this article are solely those of the authors and do not necessarily represent those of their affiliated organizations, or those of the publisher, the editors and the reviewers. Any product that may be evaluated in this article, or claim that may be made by its manufacturer, is not guaranteed or endorsed by the publisher.
