# Supplementary material for: Integrin subunit alpha V is a potent prognostic biomarker associated with immune infiltration in lower-grade glioma
Source: Front Neurol. 2022 Oct 25;13:964590. doi: 10.3389/fneur.2022.964590 (PMC9642104; doi:10.3389/fneur.2022.964590)
Supplement: Supplementary file 1 [file Table_1.DOCX]

Supplementary Material 1

##
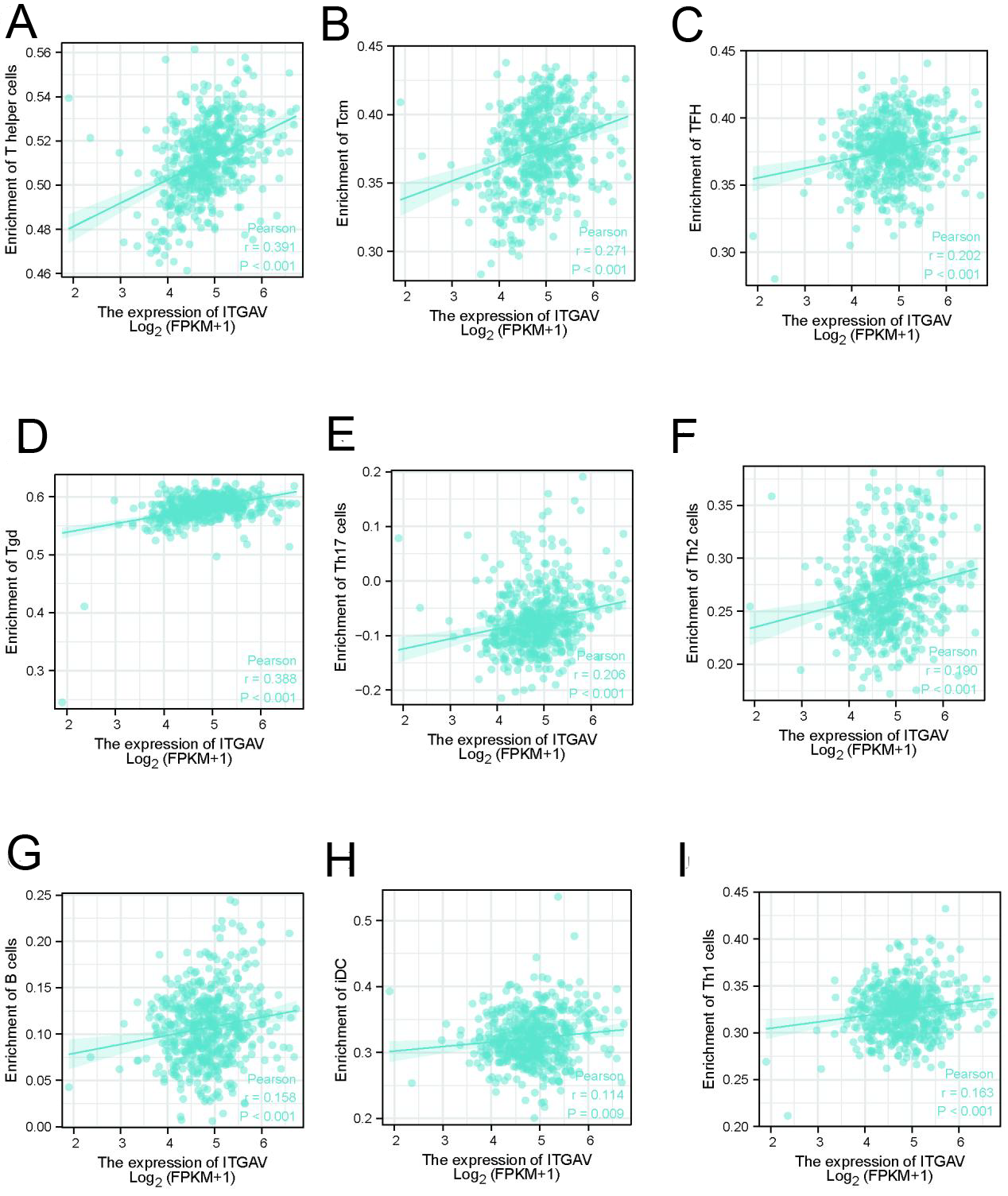


**Supplementary Figure 1.** (A-I)ITGAV expression significantly positively correlates with infiltrating levels of T helper cells，Tcm ，TFH，Tgd，Th17 cells，Th2 cells，B cells，iDC and Th1 cells.
