# Supplementary material for: Integrin subunit alpha V is a potent prognostic biomarker associated with immune infiltration in lower-grade glioma
Source: Front Neurol. 2022 Oct 25;13:964590. doi: 10.3389/fneur.2022.964590 (PMC9642104; doi:10.3389/fneur.2022.964590)
Supplement: Supplementary file 2 [file Table_2.DOCX]

Supplementary Material 2

##
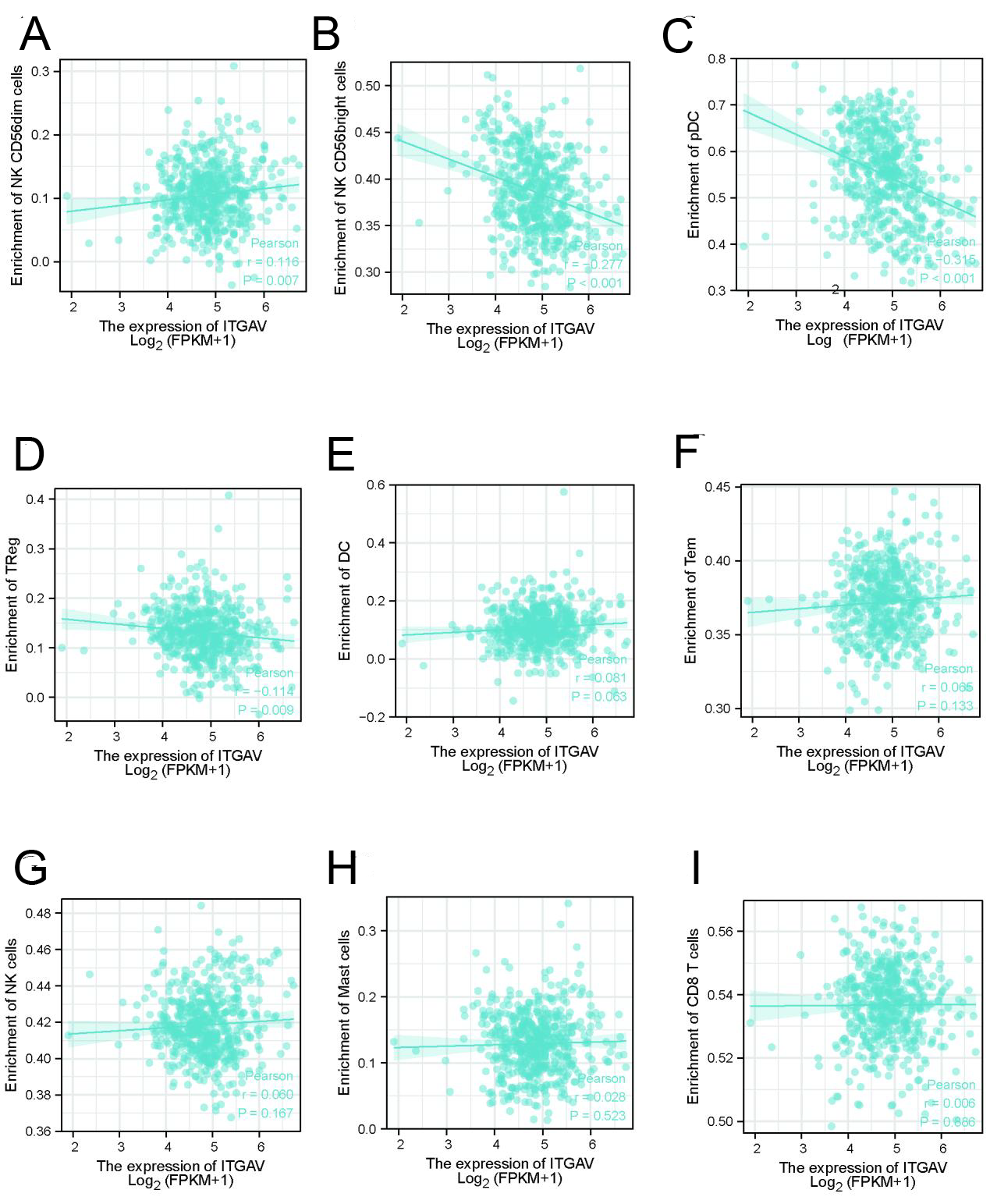


**Supplementary Figure 2.** (A-D) ITGAV expression significantly negatively correlates with infiltrating levels of CD56dim cells,CD56bright cells,pDC,TReg; (E-I) ITGAV mRNA expression was not significantly correlated with the infiltration of DC, T effector memory (Tem) , NK cells, mast cells and CD8 T cells.
